# US Policies Increase Vulnerability of Immigrant Communities to the COVID-19 Pandemic

**DOI:** 10.5334/aogh.2897

**Published:** 2020-06-10

**Authors:** Fernando A. Wilson, Jim P. Stimpson

**Affiliations:** 1 University of Utah, US; 2 Drexel University, US

## Abstract

The adverse policy environment in the United States (US) has made immigrant communities particularly vulnerable to uncontrolled community spread of COVID-19. Past and recent federal and state policy actions may exacerbate undetected community spread in immigrant communities and commensurate economic impact. Given the importance of immigrants to the US economy and society, and the human toll this pandemic is having on migrants worldwide, federal and state policies should pivot to find ways to improve access to healthcare for immigrants.

The adverse policy environment in the United States (US) has made immigrant communities particularly vulnerable to uncontrolled community spread of COVID-19. There are still very limited data on how the outbreak is impacting immigrant communities, although states and counties with high densities of immigrants also tend to have high numbers of infections, hospitalizations, and deaths [1]. Past and recent federal and state policy actions may exacerbate undetected community spread in immigrant communities and commensurate economic impact [2].

President Trump is using his emergency powers during the pandemic to push his broader agenda, which includes locking down the southern US border to severely limit immigration. This crackdown includes eschewing safeguards that protect minors and asylum seekers, allowing border control to turn them away or deport them. The stated rationale is to keep Americans safe from immigrants infected with the virus. However, there are no apparent limits on these border control tactics, leaving open the possibility that this executive order may last longer than the COVID-19 crisis and possibly throughout Trump’s term. The rationale that strict border control will limit American exposure is contradictory to what we learn from reports of immigrants in ICE detention centers testing positive for COVID-19; the overcrowded and unsanitary conditions and lack of healthcare lead to uncontained infection spread in these detention centers [3].

Immigrants to the US, particularly undocumented immigrants, face substantial economic and legal barriers to accessing healthcare. The recently passed Coronavirus Aid, Relief, and Economic Security (CARES) Act does not provide any social safety net support to the millions of undocumented immigrants. Many undocumented immigrant families live in poverty, and an equal number have no health insurance. Undocumented immigrants and most recent authorized immigrants are ineligible for federally funded public insurance programs [2]. This situation extends to most state-funded programs, even for children of immigrants; for example, most states do not provide any Medicaid benefits to pregnant immigrants if they are undocumented, less than half do not provide benefits to recently immigrated legal permanent resident (LPR) children, and nearly all states do not provide any public health insurance benefits to undocumented children [2].

Immigration policies such as the ‘public charge rule’ may further disincentivize even authorized immigrants to seek care if they develop symptoms [2]. Many immigrants from Mexico rely on medical care provided by Mexico. However, the US-Mexico border is now closed to non-essential travel. Given these factors, it is likely that more immigrants will be forced to rely on emergency departments (ED) for preventable care, thus further limiting capacity in EDs as they address COVID-19 [4]. The lack of healthcare access for immigrants, and fear of getting detained if going to the hospital, may even place US-born citizens at greater risk of infection if immigrants are unable to get tested or treated [2,4].

Compounding these issues, the US economy is expected to contract substantially. Industries in which many workers are immigrants are expected to be severely impacted [1]. Shortages of personal protective equipment (PPE) in the US will likely mean that few if any immigrant workers will have access to effective PPE for work. In addition, reduced staff in American embassies during the pandemic will slow processing of H2-A visa applications for seasonal agricultural workers. This situation may place food supply chains in jeopardy if fewer migrant workers are available for agriculture and labor shortages emerge for certain agricultural sectors. Acute hardships experienced by immigrants will likely have serious ramifications for their families and communities in their home countries. COVID-19 will be challenging for under-resourced healthcare systems in developing nations, and they are more constrained in the monetary and fiscal measures that can be implemented compared to the US, which benefits from having the US dollar as the world’s reserve currency.

The long-term impact of COVID-19 and commensurate health and economic impact on families, communities, and state and federal governments will be a topic of research for many years. As this crisis unfolds, the impact on migrants from other countries could be telling for US policy. Lack of governmental leadership, cooperation, and coordination can jeopardize lives [5]. Given the importance of immigrants to the US economy and society, and the human toll this pandemic is having on migrants worldwide, federal and state policies should pivot to find ways to improve access to healthcare for immigrants [2,3,4,5].
